# Lysosome Sensing Is a Key Mechanism in *Leishmania* Intracellular Development

**DOI:** 10.3389/fmicb.2021.667807

**Published:** 2021-05-07

**Authors:** Dan Zilberstein

**Affiliations:** Faculty of Biology, Technion – Israel Institute of Technology, Haifa, Israel

**Keywords:** *Leishmania*, development, sensing, differentiation, macrophages

## Abstract

Phagolysosomes of macrophages are the niche where the parasitic protozoan *Leishmania* resides and causes human leishmaniasis. During infection, this organism encounters dramatic environmental changes. These include heat shock (from 26°C in the vector to 33°C or 37°C in the host, for cutaneous and visceral species, respectively) and acidic pH typical to the lysosome and nutrient availability. *Leishmania* cells developed ways to sense the lysosome-specific environment (acidic pH and body temperature) as means of recognition and, subsequently, initiation of differentiation into the intracellular form. Recent studies have indicated that protein kinase A plays a role as the gatekeeper that enables differentiation initiation. This review provides an update on the lysosome signaling pathway-mediated *Leishmania* intracellular development.

## Introduction

Pathogenic microorganisms invade and colonize our body because it contains nutrients that pathogens need to complete their developmental cycle. Following infection, the microorganisms must quickly find their destinations, which is where the specific nutrients can be reached. Over many years of “learning”, these pathogens have selected host elements that are unique and thereby tag these locations. Parasites developed sensors that utilize these cues as ligands that indicate their arrival at their destination. Once they colonize, a second line of sensors monitor metabolic availability inside that compartment, enabling parasite to manipulate and survive host harsh conditions (Mancio-Silva et al., 2017; Zilberstein, 2018; Landfear and Zilberstein, 2019). My research group has investigated these processes using *Leishmania* as a model organism.

*Leishmania* is an intracellular parasite that cycles between the midgut of female sand flies and phagolysosomes of mammalian macrophages. Interestingly, *Leishmania* turned the extremely harsh environment inside the host macrophage into a shelter to most likely hide from the host immune system (Chang and Dwyer, 1976; Moradin and Descoteaux, 2012). To reach and identify the phagolysosomes, *Leishmania* developed means to sense lysosome-specific cues. Once it identifies its location inside phagolysosome, *Leishmania* starts to transform to the intracellular form, the amastigote. To block parasite invasion, macrophages activate means to kill them, including release of reactive oxygen species (ROS) and synthesis of cytotoxic nitric oxide (NO) by NO synthase (iNOS). The latter requires a massive conversion of arginine to NO, which exhausts intracellular arginine. To protect themselves, parasites developed sensing mechanism that monitor arginine in the phagolysosome, a mechanism that is essential for their survival and ability to develop into amastigotes (Goldman-Pinkovich et al., 2020).

Over almost three decades, my laboratory has investigated the signaling pathways that initiate *Leishmania* intracellular development. We developed a host-free experimental system that enabled monitoring of promastigote-to-amastigote differentiation in axenic conditions (Barak et al., 2005; Zilberstein, 2020). Using this system, we have identified parasite molecules that helped complete the puzzle of *Leishmania* development inside its host macrophage. This review is an update on this puzzle, focusing on acidic pH/high temperature-mediated differentiation. This time, I decided to tell the story with a flavor of historical perspective.

### Intracellular *Leishmania* Develops Inside Mammalian Phagolysosomes: What Does It Physiologically Mean?

It became apparent from studies in the 1970s that *Leishmania* differentiation from extracellular promastigote to intracellular amastigotes occurs within the phagolysosome of macrophages (Alexander and Vickerman, 1975; Chang and Dwyer, 1976; Berman et al., 1979). The *in vitro* studies were corroborated by the *in vivo* demonstration that when BALB/C mice footpads, infected by *Leishmania*, are suspended in a solution containing colloidal gold, the gold particles colocalized with amastigotes in the phagolysosomes of foot pad macrophages (Berman et al., 1981). These studies determined that phagocytosis is the basic mechanism underlying *Leishmania* entry into the host, but concomitant with some active input by parasites (Zilberstein and Shapira, 1994; Antoine et al., 1998; Lodge and Descoteaux, 2008).

Exactly how parasites attach to macrophages and what are the molecules that form the invasion pathway are still not fully known, even though progress has been made (Rosazza et al., 2020; Smirlis et al., 2020). A recent review has summarized genetic and biochemical details on the molecular mechanism of macrophage invasion (Horta et al., 2020). Our focus is on *Leishmania*-regulated formation of the acidic phagolysosomes and their role in the initiation of promastigote differentiation into amastigotes. During phagocytosis of uninfected macrophages, it takes about 30 min for lysosome markers to appear in a phagosome, whereas it takes more than 2 h for lysosome markers to appear in promastigote-infected phagosomes (Antoine et al., 1998; Scianimanico et al., 1999; Vinet et al., 2009; Da Silva Vieira et al., 2019). Lipophosphoglycan (LPG), a major component of the promastigote surface, plays a key role in the infective pathway as it restricts transiently the fusion of nascent promastigote-containing phagosomes with late endocytic compartments (Desjardins and Descoteaux, 1997; Moradin and Descoteaux, 2012). Interestingly, LPG delays the appearance of vacuolar ATPases in phagosome membranes until they fuse with primary lysosomes. Hence, LPG delays phagosome acidification and maturation (Vinet et al., 2009). This is critical because, as explained later, sensing acidic pH is one of the two cues that signal parasite arrival to the phagolysosome and subsequently initiate promastigote differentiation into amastigotes (Zilberstein and Shapira, 1994; Saar et al., 1998; Zilberstein, 2008).

Mutant promastigotes that lack LPG are delivered quicker to lysosomes and, consequently, are more susceptible to macrophage killing than wild-type promastigotes (Scianimanico et al., 1999; Spath et al., 2000; Turco et al., 2001; Moradin and Descoteaux, 2012). However, it is agreed by most experts in the field that LPG is not the only factor that regulates *Leishmania* entry into the host (Horta et al., 2020). For example, LPG null mutants of *Leishmania mexicana* are as effective as wild type in macrophage invasion (Ilg, 2000). The demonstration that virulent *Leishmania chagasi* is localized in caveolae during phagocytosis by host macrophages has prompted speculation that these specialized membrane domains play a role in intracellular parasite survival by targeting parasites to a phagocytosis pathway where lysosome fusion is delayed (Rodriguez et al., 2006). These analyses further support the notion that metacyclic-specific LPG delays the process of phagosome maturation until parasite complete shedding it. Hence, everything that happens after the pause is independent of LPG. Based on this model for the invasion pathway, I proposed that parasites that reach the acidic phagolysosomes and start to differentiate to amastigotes are not metacyclic anymore (Zilberstein, 2008).

To summarize the preceding section, phagolysosome biogenesis in *Leishmania*-infected macrophages is actively delayed by the invading parasite to assure that they do not initiate transformation into amastigotes before LPG is released and phagolysosome completed maturation.

### Sensing the Lysosome-Specific Environment Is How *Leishmania* Identifies Its Intracellular Destination

Once engulfed into the macrophage phagosome, except for shedding LPG, promastigotes halt development until phagosomes fuse with primary lysosomes to form the acidic phagolysosomes. Parasites that not long ago underwent heat shock (33°C and 37°C for cutaneous and visceral species, respectively) are now, in addition, exposed to acidic pH (around 5.5; Courret et al., 2001; Séguin and Descoteaux, 2016). Parasite cells combine these two cues into a single signaling pathway that indicates arrival at their destination and thereby initiates promastigote differentiation into amastigotes. However, this is not a straightforward phenomenon. It was important to show that exposing parasites concomitantly to these two stress conditions indeed activates a true signal transduction pathway, not an additional stress response.

Experiments have been carried out to prove that concomitant exposure to acidic pH and high temperature signal promastigotes to start differentiating into amastigotes. Exposing axenic promastigotes to either acidic pH without changing the temperature (Zilberstein et al., 1991) or to 37°C without changing the medium pH (Pan, 1984) induced expression of a few amastigote-specific genes, but parasites did not differentiate. Only combining both acidic pH and high temperature induced promastigote differentiation into amastigotes (Bates, 1994; Saar et al., 1998; Zilberstein, 2020). Barak et al. (2005) exposed promastigotes to pH 5.5 but replaced the high temperature with 5% methanol or 200 mM of azetidine-2-carboxylic acid, a synthetic proline analog. These compounds induce protein misfolding, typical to heat shock response. *Leishmania donovani* promastigotes grown in these conditions started to differentiate as did parasites that were exposed to the complete differentiation signal. The stronger evidence for the nature of the differentiation signal came from a phosphoproteomic experiment we have carried out in *L. donovani* promastigotes that were exposed for 2.5 h to pH 5.5 at 26°C, pH 7 at 37°C, or both, i.e., pH 5.5 at 37°C. The analysis revealed protein phosphorylation induced by either acidic pH or temperature. But most interestingly, these analyses discovered phosphorylation that was induced only by the complete signal (see figure 6 in Tsigankov et al., 2014). Eight such proteins were identified: two proteins of the translation machinery, two unknown protein kinases, and four hypothetical proteins. Altogether, these studies indicated that the combined pH 5.5 and 37°C is a true signal that activates a signal transduction pathway in promastigotes that initiate differentiation into amastigotes.

To further demonstrate that the differentiation signal induces a true signaling pathway, Rosenzweig et al. (2008) performed a proteomic time course analysis along *L. donovani* differentiation. In those days, the time affinity tagging of peptide started to merge for quantitative proteomics. My laboratory was one of the first to apply isobaric tags for relative and absolute quantitation (iTRAQ) to determine protein dynamics in differentiation. Promastigotes were exposed to the differentiation signal, and samples were collected at various time points along the 120 h of differentiation. These studies indicated that exposing promastigotes to the differentiation signal induced coordinated changes in protein abundance, including enzymes of metabolic pathways and proteins of the translation machinery (see figure 5 in Rosenzweig et al., 2008). For example, glycolytic enzyme of the cytosol gradually decreased with time as did proteins of translation pathways. In contrast, enzymes of the β oxidation pathway and amino acid catabolism gradually increased in abundance. For the first time, these iTRAQ analyses enabled to quantitate abundance changes of more >1,700 proteins at the same time point. The results of these experiments indicated that the axenic differentiation represents a highly coordinated and regulated process, a phenomenon typical to a true pathway. Further transcriptomic analyses indicated that differentiation activated dynamic changes in mRNA abundance, which suggested that at the beginning of differentiation, there were more proteins whose expression is regulated by mRNA, but later, most changes were post translational in nature (Lahav et al., 2011). In my opinion, this set of experiments clearly indicates that the differentiation signal induced a true signaling pathway that initiates promastigote differentiation into amastigotes.

Development of host-free systems using axenic parasites has enabled a better understanding of the molecular mechanism of *Leishmania* intracellular development. *L. donovani* differentiation can be induced by exposing promastigotes to high temperature and acidity (37°C, pH 5.5, 5% CO_2_) typically found in the phagolysosome (Zilberstein, 2020). Differentiation to mature amastigotes takes 5 days, resembling the time it takes *in vivo* (Courret et al., 2001). When mature axenic amastigotes are transferred into promastigote medium and incubate at 26°C, they differentiate back to promastigotes. It takes 48 h for wild-type *L. donovani* to differentiate into mature promastigotes (Bachmaier et al., 2016). This flexibility of developing back and forth enables complete control of *in vitro* analysis of the complete developmental cycle of *L. donovani*.

Barak et al. (2005) described differentiation time course and showed that following exposure to the signal, parasite cells undergo cell cycle arrest at G1. Subsequently, differentiation continues synchronously. Morphogenesis from elongated to round cells is initiated at early hours. At 12 h after exposure to the differentiation, signal parasites complete rounding; and at 24 h, they lose their flagella completely.

### Protein Kinase A Is the Differentiation Gatekeeper

Among the earliest events during promastigote-to-amastigote differentiation in *Leishmania donovani* are changes in the phosphorylation of a yet unexplored regulatory subunit of protein kinase A (LdPKAR3, LinJ.34.2680; Tsigankov et al., 2014). LdPKAR3 exists only in the genomes of intracellular trypanosomatids that include amastigotes in their life cycle, i.e., *Leishmania* and American trypanosomiasis (*Trypanosoma cruzi*). Functionally, promastigotes contain several phosphorylated proteins with PKA-specific motifs, most of them promptly dephosphorylate after initiating promastigote-to-amastigote differentiation (Bachmaier et al., 2016). These data pointed to the involvement of a PKA pathway in *Leishmania* development. My laboratory hypothesized that a check point keeps promastigotes from spontaneously transforming into amastigotes. Prompt activation of PKA dissociation opens this checkpoint, thereby initiating differentiation.

Protein kinase A is ubiquitous in eukaryotic cells, where it has been implicated in regulation of growth, development, and metabolism. The catalytic subunits of PKA assemble with the regulatory subunits into a holoenzyme complex that is inactive in the absence of cyclic AMP (cAMP). Two cyclic nucleotide-binding domains (cNBDs) in each PKAR bind cAMP and thereby cause a conformational change that leads to the dissociation of the PKAC-R complex. This unbound PKAC becomes active (Taylor et al., 2012).

*Trypanosomatid* PKAs differ from that of higher eukaryotes (Bachmaier and Boshart, 2013; Bachmaier et al., 2019); their genomes encode a regulatory subunit, PKAR1 (LinJ.13.0160 in *Leishmania*), which lacks the conserved dimerization/docking domain at the N-terminus but keeps the (pseudo) substrate inhibitor motif in the hinge and two cyclic nucleotide-binding domains at the C terminus. This ancient PKAR1 cannot dimerize, and therefore, the holoenzyme is a heterodimer, not heterotetrameric, as that of the higher eukaryotes. However, more interestingly, trypanosomatid PKA is a cAMP-independent protein kinase. Two essential arginines in their cNBD-binding pockets contain other amino acids (Bubis et al., 2018; Bachmaier et al., 2019). Evolutionarily, this indicates that PKAR underwent significant changes in higher single-cell Eukaryotes (i.e., plasmodium and yeast) until it became a cAMP-dependent protein kinase. This hypothesis is supported by the fact that genomes of the trypanosomatid family lack the G-trimeric proteins (Ivens et al., 2005; Landfear and Zilberstein, 2019). These findings indicate that the older versions of PKAR in which trypanosomatids regulate PKAC activities are different from the canonical PKA.

In addition to R1, *Leishmania* genome encodes LdPKAR3 (LinJ.34.2680) that similar to R1 form heterodimers with LdPKAC subunits and is cAMP-independent. In LdPKAR3, the C-terminus half is conserved with higher Eukaryotes while the N-terminus half is divergent and most likely unstructured (Fischer-Weinberger et al., 2021).

A salient feature of the R3 subunit is a set of 12 phosphorylation sites that dynamically change during differentiation (Tsigankov et al., 2013, 2014). Of these, there was a four-fold increase in the phosphorylation of serine 262 (S262) within minutes of exposure to the promastigote-to-amastigote differentiation signal. Phosphorylation of this site is also achieved by exposure to acidic pH only, supporting an idea that this phosphorylation is induced by a pH sensor that activates a downstream kinase that subsequently phosphorylates S262. This site localizes within a region of LdPKAR3 that is likely to interact with a catalytic subunit (LdPKAC).

*Leishmania donovani* (as do all *Leishmania* species) has three distinct catalytic subunits (LdPKAC1, LdPKAC2, and LdPKAC3, encoded by *LinJ.35.4060*, *LinJ.35.4010*, and *LinJ.18.1090*, respectively). Extensive phylogenetic analyses indicated that LdPKAC3 is conserved across all taxa, while C1 and C2 are restricted to the Kinetoplastidae. Recent experiments indicate that LdPKAR3 is covalently bound to the subpellicular microtubules at the cell cortex. R3 associates with C3, and this association is important for the elongated shape of promastigotes (Fischer-Weinberger et al., 2021).

Cumulatively, *Leishmania* promastigotes have the means to sense the lysosome environment, and together with heat shock response, they turn these cues to a signal that initiates differentiation. Moreover, to date, data support the idea that *Leishmania* PKA plays a role in transducing promastigote-to-amastigote differentiation signal, thereby initiating differentiation.

### Iron Metabolism Activates an Alternative *Leishmania* Differentiation Pathway

Even though this review focuses on acidic pH/high temperature-induced differentiation, it is not the only pathway described to date. It is established that stress, mostly deprivation of essential metabolites, triggers parasites to escape to a life form that is less susceptible to these stresses. Amastigotes are “stress-relaxed” organisms. For example, heat (Pan, 1984), overflow of serum (Doyle et al., 1991), and osmotic shock (Blum and Balber, 1996) impose shape change in promastigotes. None of these stresses induced complete differentiation of promastigotes into amastigotes, except for the stress induced by iron. The laboratory of Norma Andrews has investigated iron transport and metabolism in *Leishmania* and its role in virulence and development. They found a novel role for iron uptake in orchestrating the differentiation of amastigotes, through a mechanism that involves production of ROS (Mittra and Andrews, 2013) and is independent from pH and temperature changes. ROS are generally thought to be deleterious for pathogens, but it is becoming increasingly apparent that they can also function as signaling molecules regulating *Leishmania* differentiation, in a process that is tightly controlled by iron availability. These studies indicated that the ability to import iron is critical for both promastigotes and amastigotes viability. Interestingly, *Leishmania* ferric iron reductase 1 (LFR1) overexpression induced differentiation into amastigote-like parasites (Rocco-Machado et al., 2019). These cells resembled intracellular amastigotes as they are round, lost most of their flagella, and are virulent. How this iron-dependent pathway functions in disease development is still to be investigated.

## Concluding Remarks

In 2001, Burchmore and Barrett published an article titled “Life in vacuoles – nutrient acquisition by *Leishmania* amastigotes” where they described what was known almost 20 years ago on how *Leishmania* parasite metabolically co-op with the extreme environment inside the phagolysosome (Burchmore and Barrett, 2001). The focus was on how nutrient transport and metabolism are influenced by the acidic pH environment encountered by amastigotes. A few years earlier, Shapira and Zilberstein speculated that acidic pH and high temperature are key elements in signaling promastigotes’ arrival at their destination and to employ means of adaptation to the phagolysosome milieu. Our laboratory is getting close to describe the first amino acid-sensing signal transduction pathway. This pathway will open new avenues to understand intracellular parasitism.

## Author Contributions

The author confirms being the sole contributor of this work and has approved it for publication.

## Conflict of Interest

The author declares that the research was conducted in the absence of any commercial or financial relationships that could be construed as a potential conflict of interest.

## References

[B1] Alexander J. Vickerman K. (1975). Fusion of host cell secondary lysosomes with the parasitophorous vacuoles of *Leishmania mexicana*-infected macrophages. *J. Protozool.* 22 502–508. 10.1111/j.1550-7408.1975.tb05219.x 172627

[B2] Antoine J. C. Prina E. Lang T. Courret N. (1998). The biogenesis and properties of the parasitophorous vacuoles that harbour *Leishmania* in murine macrophages. *Trends Microbiol.* 6 392–401. 10.1016/s0966-842x(98)01324-9 9807783

[B3] Bachmaier S. Boshart M. (2013). “Kinetoplastid AGC kinases,” in *Protein Phosphorylation in Eukaryotic Parasites: Potential for Chemotherapy*, eds Doerig D. Spaeth C. S. Wiese M. Selzer P. M. (Hoboken, NJ: Wiley-Blackwell).

[B4] Bachmaier S. Volpato Santos Y. Kramer S. Githure G. B. Klöckner T. Pepperl J. (2019). Nucleoside analogue activators of cyclic AMP-independent protein kinase A of *Trypanosoma*. *Nat. Commun.* 10:1421. 10.1038/s41467-019-09338-z 30926779 PMC6440977

[B5] Bachmaier S. Witztum R. Tsigankov P. Koren R. Boshart M. Zilberstein D. (2016). Protein kinase A signaling during bidirectional axenic differentiation in *Leishmania*. *Int. J. Parasitol.* 46 75–82. 10.1016/j.ijpara.2015.09.003 26460237

[B6] Barak E. Amin-Spector S. Gerliak E. Goyard S. Holland N. Zilberstein D. (2005). Differentiation of *Leishmania donovani* in host-free system: analysis of signal perception and response. *Mol. Biochem. Parasitol.* 141 99–108. 10.1016/j.molbiopara.2005.02.004 15811531

[B7] Bates P. A. (1994). Complete developmental cycle of *Leishmania mexicana* in axenic culture. *Parasitology* 108(Pt. 1), 1–9. 10.1017/s0031182000078458 8152848

[B8] Berman J. D. Fioretti T. B. Dwyer D. M. (1981). In vivo and in vitro localization of Leishmania within macrophage phagolysosomes: use of colloidal gold as a lysosomal label. *J. Protozool.* 28 239–242. 10.1111/j.1550-7408.1981.tb02839.x 7277257

[B9] Berman J. D. Wyler D. J. Dwyer D. M. (1979). Multiplication of *Leishmania* in human macrophages in vitro. *Infect.Immun.* 26 375–379. 10.1128/iai.26.1.375-379.1979 500212 PMC414622

[B10] Blum J. J. Balber A. E. (1996). Osmotic and metabolic-induced changes in light scattering of *Leishmania donovani* as measured by flow cytometry. *J. Eukaryot. Microbiol.* 43 213–217. 10.1111/j.1550-7408.1996.tb01393.x

[B11] Bubis J. Martinez J. C. Calabokis M. Ferreira J. Sanz-Rodriguez C. E. Navas V. (2018). The gene product of a *Trypanosoma equiperdum* ortholog of the cAMP-dependent protein kinase regulatory subunit is a monomeric protein that is not capable of binding cyclic nucleotides. *Biochimie* 146 166–180. 10.1016/j.biochi.2017.12.010 29288679 PMC6550279

[B12] Burchmore R. J. Barrett M. P. (2001). Life in vacuoles–nutrient acquisition by *Leishmania* amastigotes. *Int. J. Parasitol.* 31 1311–1320. 10.1016/s0020-7519(01)00259-4 11566299

[B13] Chang K. P. Dwyer D. M. (1976). Multiplication of a human parasite (*Leishmania donovani*) in phagolysosomes of hamster macrophages in vitro. *Science* 193 678–680. 10.1126/science.948742 948742

[B14] Courret N. Frehel C. Prina E. Lang T. Antoine J. C. (2001). Kinetics of the intracellular differentiation of *Leishmania amazonensis* and internalization of host MHC molecules by the intermediate parasite stages. *Parasitology* 122 263–279. 10.1017/s0031182001007387 11289063

[B15] Da Silva Vieira T. Arango Duque G. Ory K. Gontijo C. M. Soares R. P. Descoteaux A. (2019). *Leishmania braziliensis*: strain-specific modulation of phagosome maturation. *Front. Cell. Infect. Microbiol.* 9:319. 10.3389/fcimb.2019.00319 31555609 PMC6743224

[B16] Desjardins M. Descoteaux A. (1997). Inhibition of phagolysosomal biogenesis by the *Leishmania* lipophosphoglycan. *J. Exp. Med.* 185 2061–2068. 10.1084/jem.185.12.2061 9182677 PMC2196352

[B17] Doyle P. S. Engel J. C. Pimenta P. F. Da Silva P. P. Dwyer D. M. (1991). *Leishmania donovani*: long-term culture of axenic amastigotes at 37 degrees C. *Exp. Parasitol.* 73 326–334. 10.1016/0014-4894(91)90104-5 1915747

[B18] Fischer-Weinberger R. Bachmaier S. Dandugudumula R. Phan I. A. Almoznino M. Githur G. B. (2021). A divergent protein kinase A in the human pathogen *Leishmania* is associated with developmental morphogenesis. *bioRxiv*. 10.1101/2021.04.24.440790 PMC11006142 38551993

[B19] Goldman-Pinkovich A. Kannan S. Nitzan-Koren R. Puri M. Pawar H. Bar-Avraham Y. (2020). Sensing host arginine is essential for *Leishmania* parasites’ intracellular development. *MBio* 11:e02023–20. 33051367 10.1128/mBio.02023-20 PMC7554669

[B20] Horta M. F. Andrade L. O. Martins-Duarte ÉS. Castro-Gomes T. (2020). Cell invasion by intracellular parasites - the many roads to infection. *J. Cell Sci.* 133:jcs232488. 10.1242/jcs.232488 32079731

[B21] Ilg T. (2000). Lipophosphoglycan is not required for infection of macrophages or mice by *Leishmania mexicana*. *EMBO J.* 19 1953–1962. 10.1093/emboj/19.9.1953 10790362 PMC305689

[B22] Ivens A. C. Peacock C. S. Worthey E. A. Murphy L. Aggarwal G. Berriman M. (2005). The genome of the kinetoplastid parasite, *Leishmania major*. *Science* 309 436–442. 10.1126/science.1112680 16020728 PMC1470643

[B23] Lahav T. Sivam D. Volpin H. Ronen M. Tsigankov P. Green A. (2011). Multiple levels of gene regulation mediate differentiation of the intracellular pathogen *Leishmania*. *FASEB J.* 25 515–525. 10.1096/fj.10-157529 20952481 PMC6188352

[B24] Landfear S. M. Zilberstein D. (2019). Sensing what’s out there - kinetoplastid parasites. *Trends Parasitol.* 35 274–277. 10.1016/j.pt.2018.12.004 30655057 PMC7392150

[B25] Lodge R. Descoteaux A. (2008). *Leishmania* invasion and phagosome biogenesis. *Subcell. Biochem.* 47 174–181. 10.1007/978-0-387-78267-6_14 18512351

[B26] Mancio-Silva L. Slavic K. Grilo Ruivo M. T. Grosso A. R. Modrzynska K. K. Vera I. M. (2017). Nutrient sensing modulates malaria parasite virulence. *Nature* 547 213–216. 10.1038/nature23009 28678779 PMC5511512

[B27] Mittra B. Andrews N. W. (2013). IRONy OF FATE: role of iron-mediated ROS in *Leishmania* differentiation. *Trends Parasitol.* 29 489–496. 10.1016/j.pt.2013.07.007 23948431 PMC3783550

[B28] Moradin N. Descoteaux A. (2012). *Leishmania* promastigotes: building a safe niche within macrophages. *Front. Cell Infect. Microbiol.* 2:121. 10.3389/fcimb.2012.00121 23050244 PMC3445913

[B29] Pan A. A. (1984). Leishmania mexicana?: serial cultivation of intracellular stages in a cell-free medium. *Exp. Parasitol.* 58 72–80. 10.1016/0014-4894(84)90022-5 6745388

[B30] Rocco-Machado N. Cosentino-Gomes D. Nascimento M. T. Paes-Vieira L. Khan Y. A. Mittra B. (2019). Leishmania amazonensis ferric iron reductase (LFR1) is a bifunctional enzyme: unveiling a NADPH oxidase activity. *Free Radic. Biol. Med.* 143 341–353. 10.1016/j.freeradbiomed.2019.08.026 31446054

[B31] Rodriguez N. E. Gaur U. Wilson M. E. (2006). Role of caveolae in *Leishmania chagasi* phagocytosis and intracellular survival in macrophages. *Cell Microbiol.* 8 1106–1120. 10.1111/j.1462-5822.2006.00695.x 16819964

[B32] Rosazza T. Lecoeur H. Blisnick T. Moya-Nilges M. Pescher P. Bastin P. (2020). Dynamic imaging reveals surface exposure of virulent *Leishmania* amastigotes during pyroptosis of infected macrophages. *J. Cell Sci.* 134 jcs242776. 10.1242/jcs.242776 32501279

[B33] Rosenzweig D. Smith D. Opperdoes F. Stern S. Olafson R. W. Zilberstein D. (2008). Retooling *Leishmania* metabolism: from sand fly gut to human macrophage. *FASEB J.* 22 590–602. 10.1096/fj.07-9254com 17884972

[B34] Saar Y. Ransford A. Waldman E. Mazareb S. Amin-Spector S. Plumblee J. (1998). Characterization of developmentally-regulated activities in axenic amastigotes of *Leishmania donovani*. *Mol. Biochem. Parasitol.* 95 9–20. 10.1016/s0166-6851(98)00062-0 9763285

[B35] Scianimanico S. Desrosiers M. Dermine J. F. Meresse S. Descoteaux A. Desjardins M. (1999). Impaired recruitment of the small GTPase rab7 correlates with the inhibition of phagosome maturation by *Leishmania donovani* promastigotes. *Cell Microbiol.* 1 19–32. 10.1046/j.1462-5822.1999.00002.x 11207538

[B36] Séguin O. Descoteaux A. (2016). *Leishmania*, the phagosome, and host responses: the journey of a parasite. *Cell. Immunol.* 309 1–6. 10.1016/j.cellimm.2016.08.004 27531526

[B37] Smirlis D. Dingli F. Pescher P. Prina E. Loew D. Rachidi N. (2020). SILAC-based quantitative proteomics reveals pleiotropic, phenotypic modulation in primary murine macrophages infected with the protozoan pathogen *Leishmania donovani*. *J. Proteom.* 213:103617. 10.1016/j.jprot.2019.103617 31846769

[B38] Spath G. F. Epstein L. Leader B. Singer S. M. Avila H. A. Turco S. J. (2000). Lipophosphoglycan is a virulence factor distinct from related glycoconjugates in the protozoan parasite *Leishmania major*. *Proc. Natl. Acad. Sci. U.S.A.* 97 9258–9263. 10.1073/pnas.160257897 10908670 PMC16855

[B39] Taylor S. S. Ilouz R. Zhang P. Kornev A. P. (2012). Assembly of allosteric macromolecular switches: lessons from PKA. *Nat. Rev. Mol. Cell Biol.* 13 646–658. 10.1038/nrm3432 22992589 PMC3985763

[B40] Tsigankov P. Gherardini P. F. Helmer-Citterich M. Spaeth G. F. Myler P. J. Zilberstein D. (2014). Regulation dynamics of *Leishmania* differentiation: deconvoluting signals and identifying phosphorylation trends. *Mol. Cell Proteom.* 13 1769–1786. 10.1074/mcp.M114.037705 24741111 PMC4083115

[B41] Tsigankov P. Gherardini P. F. Helmer-Citterich M. Spath G. F. Zilberstein D. (2013). Phosphoproteomic analysis of differentiating *Leishmania* parasites reveals a unique stage-specific phosphorylation motif. *J. Proteome Res.* 12 3405–3412. 10.1021/pr4002492 23688256

[B42] Turco S. J. Spath G. F. Beverley S. M. (2001). Is lipophosphoglycan a virulence factor? A surprising diversity between Leishmania species. *Trends Parasitol.* 17 223–226. 10.1016/s1471-4922(01)01895-5 11323305

[B43] Vinet A. F. Fukuda M. Turco S. J. Descoteaux A. (2009). The *Leishmania donovani* lipophosphoglycan excludes the vesicular proton-ATPase from phagosomes by impairing the recruitment of synaptotagmin V. *PLoS Pathog.* 5:e1000628. 10.1371/journal.ppat.1000628 19834555 PMC2757729

[B44] Zilberstein D. (2008). “Physiological and biochemical aspects of leishmania develpment,” in *Leishmania After The genome: Biology and Control*, eds Myler P. J. Fasel N. (New York, NY: Horizon Scientific Press), 107–122.

[B45] Zilberstein D. (2018). “Nutrient transport and sensing as pharmacological targets for leishmaniasis,” in *Drug Discovery for Leishmaniasis*, eds Rivas L. Carmen G. (London: The Royal Society of Chemistry), 282–296. 10.1039/9781788010177

[B46] Zilberstein D. (2020). In vitro culture for differentiation simulation of *Leishmania* spp. *Methods Mol. Biol.* 2116 39–47. 10.1007/978-1-0716-0294-2_3 32221912

[B47] Zilberstein D. Shapira M. (1994). The role of pH and temperature in the development of *Leishmania* parasites. *Annu. Rev. Microbiol.* 48 449–470. 10.1146/annurev.mi.48.100194.002313 7826014

[B48] Zilberstein D. Blumenfeld N. Liveanu V. Gepstein A. Jaffe C. L. (1991). Growth at acidic pH induces an amastigote stage-specific protein in *Leishmania* promastigotes. *Mol. Biochem. Parasitol.* 45 175–178. 10.1016/0166-6851(91)90040-d 2052037

